# Germanene-Based Two-Dimensional Magnet with Tunable Properties

**DOI:** 10.1021/acsnano.5c03331

**Published:** 2025-05-29

**Authors:** Andrey V. Matetskiy, Alessandro Barla, Paolo Moras, Carlo Carbone, Valeria Milotti, Carlo Alberto Brondin, Zipporah Rini Benher, Mariia Holub, Philippe Ohresser, Edwige Otero, Fadi Choueikani, Igor A. Shvets, Alexey N. Mihalyuk, Sergey V. Eremeev, Polina M. Sheverdyaeva

**Affiliations:** † CNR-Istituto di Struttura della Materia (CNR-ISM), Strada Statale 14, km 163.5, 34149 Trieste, Italy; ‡ Synchrotron-SOLEIL, L’Orme des Merisiers, 91190 Saint-Aubin , France; § Tomsk State University, Tomsk 634050, Russia; ∥ Institute of High Technologies and Advanced Materials, Far Eastern Federal University, Vladivostok 690950, Russia; ⊥ Institute of Automation and Control Processes FEB RAS, Vladivostok 690041, Russia; # Institute of Strength Physics and Materials Science SB RAS, Tomsk 634055, Russia; ∇ 28334 Peter Grünberg Institute (PGI-3), 52428 Forschungszentrum Jülich, Germany; ○ Dipartimento di Fisica e Astronomia “Galileo Galilei”, Università degli Studi di Padova, 35122 Padova, Italy; ▲ St. Petersburg State University, 7/9 Universitetskaya nab., St. Petersburg 199034, Russia

**Keywords:** germanene, 2D materials, ARPES, DFT, reversible AFM-FM transition

## Abstract

Magnetic order engineering in two-dimensional Dirac systems is of great interest for theoretical and technological exploration. Up to now, the experimental advances in this field mostly concerned graphene monolayers. Here, we report a comprehensive study of a monolayer-thick germanene-like sheet in contact with gadolinium atoms. Direct observations supported by first-principles calculations reveal the fingerprints of the Dirac fermions in the electronic structure and noncollinear antiferromagnetism. The hybridization of the germanene layer with Gd atoms leads to a large and tunable gap in the Dirac states that carry a nonzero spin-Berry curvature. We discovered that cesium-induced controlled electron doping can switch the system into a ferromagnetic state and then back to the antiferromagnetism at saturated cesium monolayer limit. We explain these reversible magnetic transitions by the oscillatory behavior of the Ruderman–Kittel–Kasuya–Yosida interaction and suggest that this system could find application in magnetoelectronics and spintronics.

## Graphical abstract

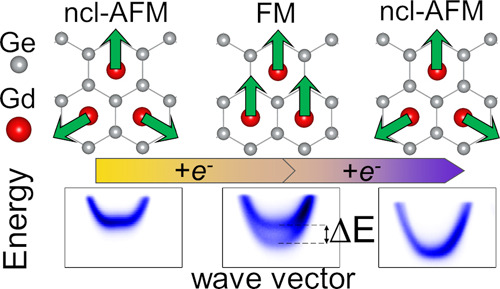

Two-dimensional (2D) magnetic materials are of interest for the field of magnetism and spintronics as well as for the 2D materials community. They allow for the fundamental studies of magnetism in the 2D limit and are promising for device applications based on the integration of 2D magnetic layers. Although many theoretical works predicted a large amount of possible magnetic monolayers,^1–3^ there is a limited number of experimental reports. Intrinsic magnetism has been experimentally reported in the 3*d* metal atoms based materials such as Cr_2_Ge_2_Te_6_,^4^ CrI_3_,^5^ VSe_2_,^6^ showing ferromagnetic (FM) order, and in FePS_3_,^7^ MnPS_3_,^8^ MnPSe_3_ ^9^ with antiferromagnetic (AFM) order. The magnetic order of the 2D magnetic materials can be easily tuned by surface doping,^10^ electric field^4^ and strain.^11^ Magnetism has also been induced in nonmagnetic monoelemental monolayers such as graphene by proximity to a magnetic element, a substrate^12^ or magnetic adatoms.^13^ Compared to the 3*d* elements, the rare-earth (RE) metals offer a new interesting scenario, as they possess partially filled and highly localized 4*f* shells leading to large magnetic moments.^14^ The Ruderman-Kittel-Kasuya-Yosida (RKKY) interaction depends on the conduction electrons and leads to oscillatory behavior between FM and AFM.^15^ For 2D materials, this mechanism was predicted to allow an efficient tuning of the exchange interaction by modifying the chemical potential.^16,17^ The large variety of RE metals with similar structural parameters but with different valences and spin and orbital magnetic moments offers an interesting playground for studying magnetism in 2D systems.^18,19^

One of the important goals for the engineering of a 2D material is to find an appropriate supporting substrate. This should be semiconducting, and possibly have a similar lattice parameter. In such a system one can limit the hybridization of the 2D states with the bulk, confining the electrons in the monolayer and enhancing effects of surface doping. At the same time, this would open the way for the integration of the 2D magnet into real devices. The GdGe_2_ monolayer, grown by Gd intercalation on Ge(111)^20,21^ combines buckled germanene atomic structure,^22^ large magnetic moments of Gd,^23–25^ and a semiconducting substrate. Compared with graphene and silicene, germanene was poorly addressed experimentally in the monolayer limit, despite having interesting electronic properties.^26^ Namely, it is predicted to have a large and tunable gap in the ππ* Dirac cones and nontrivial topological properties.^27^ The unoccupied band structure of germanene hosts peculiar heavy fermionic states sensitive to perturbations and located much closer to the Fermi level (*E*_F_) as compared to graphene and silicene.^28,29^ Hence, the new findings call for a dedicated study of the electronic and magnetic structure of the GdGe_2_ monolayer, which so far has been only partially addressed.^20,21,23,24^

Here we report on an angle-resolved photoemission spectroscopy (ARPES) and X-ray magnetic circular dichroism (XMCD) study complemented by accurate density functional theory (DFT) calculations of a germanene-based GdGe_2_ monolayer. ARPES data, in agreement with our DFT calculations, demonstrate the unambiguous formation of heavily doped gapped Dirac cones and massive van Hove singularities (vHSs), both located in the substrate’s bulk band gap. Large electron doping brings to the Fermi level new singularity states, which allow to realize multiple electronic and magnetic transitions. In-situ XMCD magnetic characterization and DFT calculations revealed the noncollinear AFM (ncl-AFM) magnetic state in the GdGe_2_ monolayer. We demonstrate that controlled electron doping by adsorption of cesium can switch this magnetic order into a FM one with an exchange splitting of the bands near *E*_F_ clearly observable in ARPES. However, the formation of a saturated Cs layer drives the system back to the ncl-AFM state. We explain our findings by the oscillatory behavior of the RKKY interaction in a 2D magnet.^16,17,30^ We suggest that the realized system is an important 2D model material with tunable electronic and magnetic properties.

## Results and Discussion

### Dirac Fermions in GdGe_2_: Electronic Properties and Topology

The ordering and symmetry of the 1 ML GdGe_2_ film, which is a buckled germanene-like layer with Gd atoms centered in the voids^24^ (Figure 1a, see also detailed atomic structure in Supplementary Figure S1), were characterized by LEED measurements, as shown in Figure 1b. The six sharp hexagonal spots in the LEED pattern correspond to the Ge(111)-(1 × 1) lattice, indicating ideal matching between the formed film and the substrate. This lattice match avoids the umklapp replica of substrate’s valence states that often hinders the interpretation of ARPES data in similar materials.^31,32^ The sharp appearance of the LEED pattern suggests the formation of a homogeneous GdGe_2_ monolayer.

**Figure 1:**
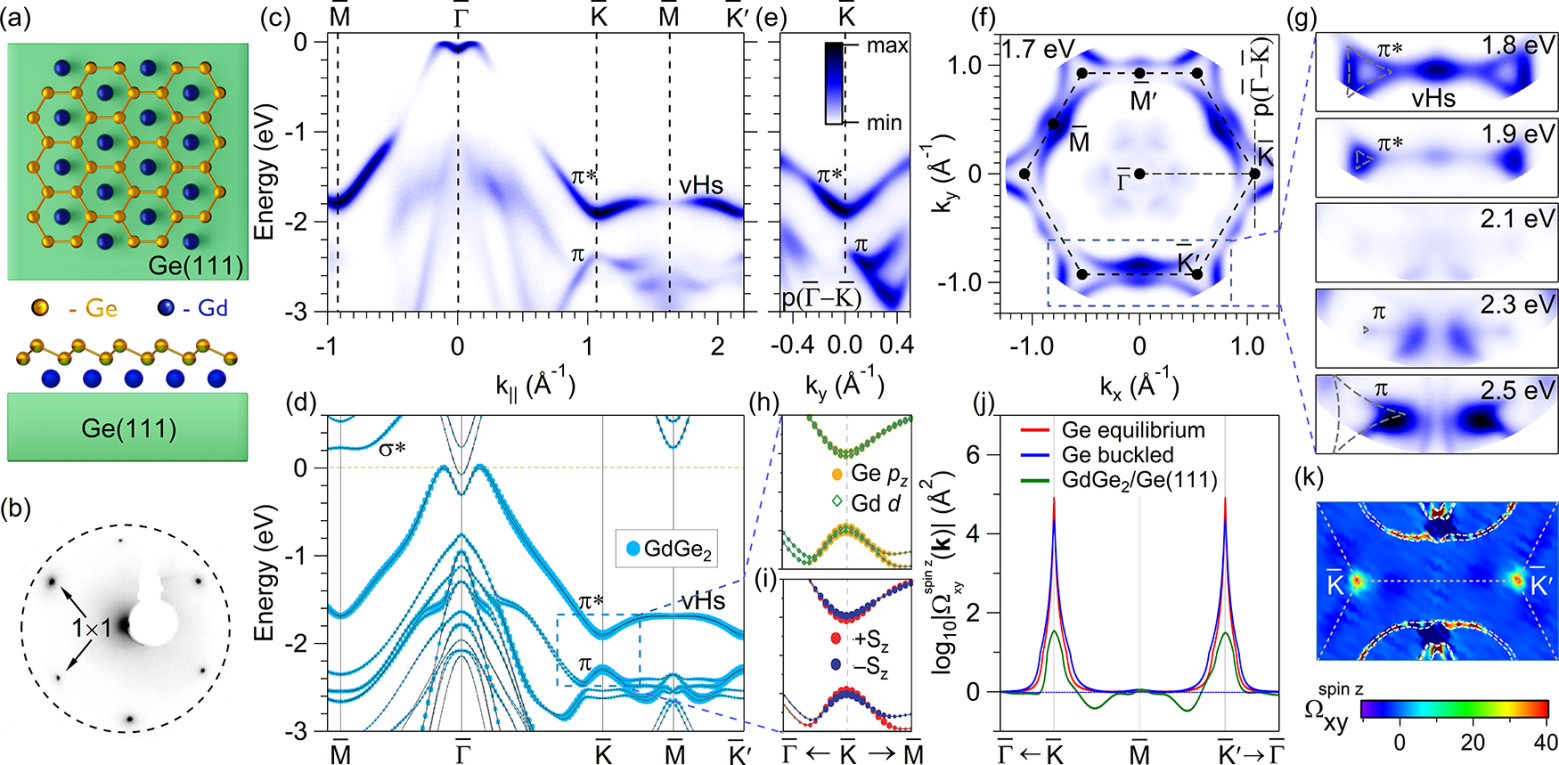
Electronic and topological properties of the GdGe_2_monolayer. (a) Atomic structure of the GdGe_2_ monolayer on the Ge(111) substrate in top and side view and its (b) LEED pattern with 1 × 1 reflexes marked by arrows. (c) ARPES spectrum along M̅-Γ̅-K̅-M̅-K̅*′* high-symmetry directions taken at 14 K with 35 eV photon energy. (d) Calculated spectra of nonmagnetic phase of GdGe_2_/Ge­(111). Size of blue circles corresponds to the weight of the states localized in the GdGe_2_ layer. (e) ARPES spectrum along *p*(Γ̅-K̅). (f) Constant energy map taken at a binding energy of 1.7 eV. (g) Evolution of the constant energy map close to the K̅-M̅-K̅′ line as a function of the binding energy. The dashed lines highlight the contours of the π and π* bands. Details of the K̅ point Dirac state with (h) orbital and (i) out-of-plane (*S*_*z*_) spin projections. (j) Calculated spin Berry curvature (in logarithmic scale) in the vicinity of K̅ and K̅*′* for equilibrium (low-buckled) and buckled germanene (as it separated from the GdGe_2_) and substrate-supported GdGe_2_ monolayer. (k) Spin-Berry curvature map around K̅-M̅-K̅′ path, assuming the *E*_F_ within the local K̅ gap.

Figure 1c shows the low-temperature (14 K) ARPES spectrum along M̅-Γ̅-K̅-M̅-K̅′, which is identical to high-temperature (82 K) ARPES map except for thermal broadening (see Supplementary Figure S2). Two intense features marked as π and π* bands dominate the spectra and are absent in the ARPES intensity map of the clean Ge(111) surface (see Supplementary Figure S3). The highly intense appearance indicates surface localization of these bands. Figure 1d shows the theoretical band structure (calculated using the hybrid functional HSE06), which is in perfect agreement with the ARPES spectrum (see also Supplementary Figure S4). We can identify the two above-mentioned bands as analogs of the π and π* bands of a buckled 2D honeycomb lattice, shifted deep below *E*_F_ by electron doping. The calculations predict two nondegenerate spin branches for each band; however, this splitting is too small to be observed in ARPES data. The π* state lies completely in the substrate’s gap, approaching *E*_F_ near Γ̅ and reaching a minimum at K̅. The π band strongly overlaps with the substrate states, except for the proximity of K̅ where it reaches a maximum. A gapped Dirac cone formed by the π and π* states is clearly visible at K̅ at ≈2 eV below *E*_F_ (Figure 1e). Figure 1e shows the direction perpendicular to Γ̅-K̅ (see Figure 1f for the schematics of the directions). From here we estimated a gap width of 0.5 eV. Along Γ̅-K̅-M̅ the π* band shows a highly anisotropic slope, that strongly decreases toward M̅ (Figure 1c). The so-formed band at M̅ is the vHS of germanene. Its bandwidth (0.12 eV) is much smaller compared to that of graphene (about 1.5 eV) and of free-standing germanene (1.0 eV).^22^ As a result, a straight contour can be observed at a binding energy of 1.7 eV together with triangular contours of the π* band (Figure 1f). Figure 1g shows constant energy cuts with a focus on the K̅-M̅-K̅*′* line as a function of the binding energy. Similar constant energy cuts were reported for the Lifshitz transition in overdoped graphene.^33^

The gap magnitude of 0.5 eV at K̅ is among the largest predicted or observed so far in germanene systems.^34,35^ This is an order of magnitude larger than in the free-standing equilibrium state or in germanene with substrate-induced buckling (see Supplementary Figure S5), where the gap has a spin–orbit coupling origin. In the GdGe_2_ film it originates primarily from the hybridization of Ge-*p*_*z*_ and Gd-*d* orbitals (Figure 1h). Furthermore, this hybridization also results in a lifting of the spin degeneracy in the Dirac state mentioned above. Figure 1i shows the zoom on the Dirac point, where the out-of-plane spin polarization is shown.

The gap in free-standing germanene was predicted to have nontrivial topological properties.^27^ The calculated spin Berry curvature for free-standing equilibrium (low-buckled) germanene (Figure 1j, red line) has a δ-function-like distribution of the same positive sign in the vicinity of K̅(K̅*′*) resulting in nontrivial *Z*_2_ = 1 topological invariant, therefore proving the quantum spin Hall (QSH) phase. While for equilibrium germanene *E*_F_ is naturally located within the SOC-driven gap, in the free-standing Ge bilayer with high-buckled lattice, as induced by the Gd layer, it shifts about ∼0.5 eV higher (Supplementary Figure S5). However, assuming for the latter case that *E*_F_ lies within the local K̅ gap, the spin Berry curvature becomes smaller in magnitude and isotropically spreads more around K̅ and K̅*′* (Figure 1j, blue line). Finally, in the Dirac state of the GdGe_2_/Ge­(111) the calculated distribution of the local spin Berry curvature is still persistent, though it becomes less pronounced, and remarkably anisotropic (Figure 1j,k). Thus, the GdGe_2_ monolayer, although it is not a QSH insulator, still inherits the local nonzero spin Berry curvature – the feature of the Dirac state of the free-standing germanene.

### Tuning the Electronic Properties of GdGe_2_

It has been predicted that an electric field or doping can vary the Dirac cone gap.^27,34^ In order to investigate these predictions, we performed a controlled electron doping of the GdGe_2_ film through Cs adsorption. Indeed, the energy position of the Dirac state, the K̅-gap width, and the width of the van Hove singularity can be effectively tuned by electron doping (Figure 2a). The π–π* gap decreases and reaches 0.32 eV for saturated Cs doping (1 ML). The slope of the Dirac state along K̅ – Γ̅ and the bandwidth of the vHS increase, resembling more the vHS of the free-standing germanene. Under a short annealing, the effects of Cs doping can be removed, making the system largely reversible (Supplementary Figure S6).

**Figure 2:**
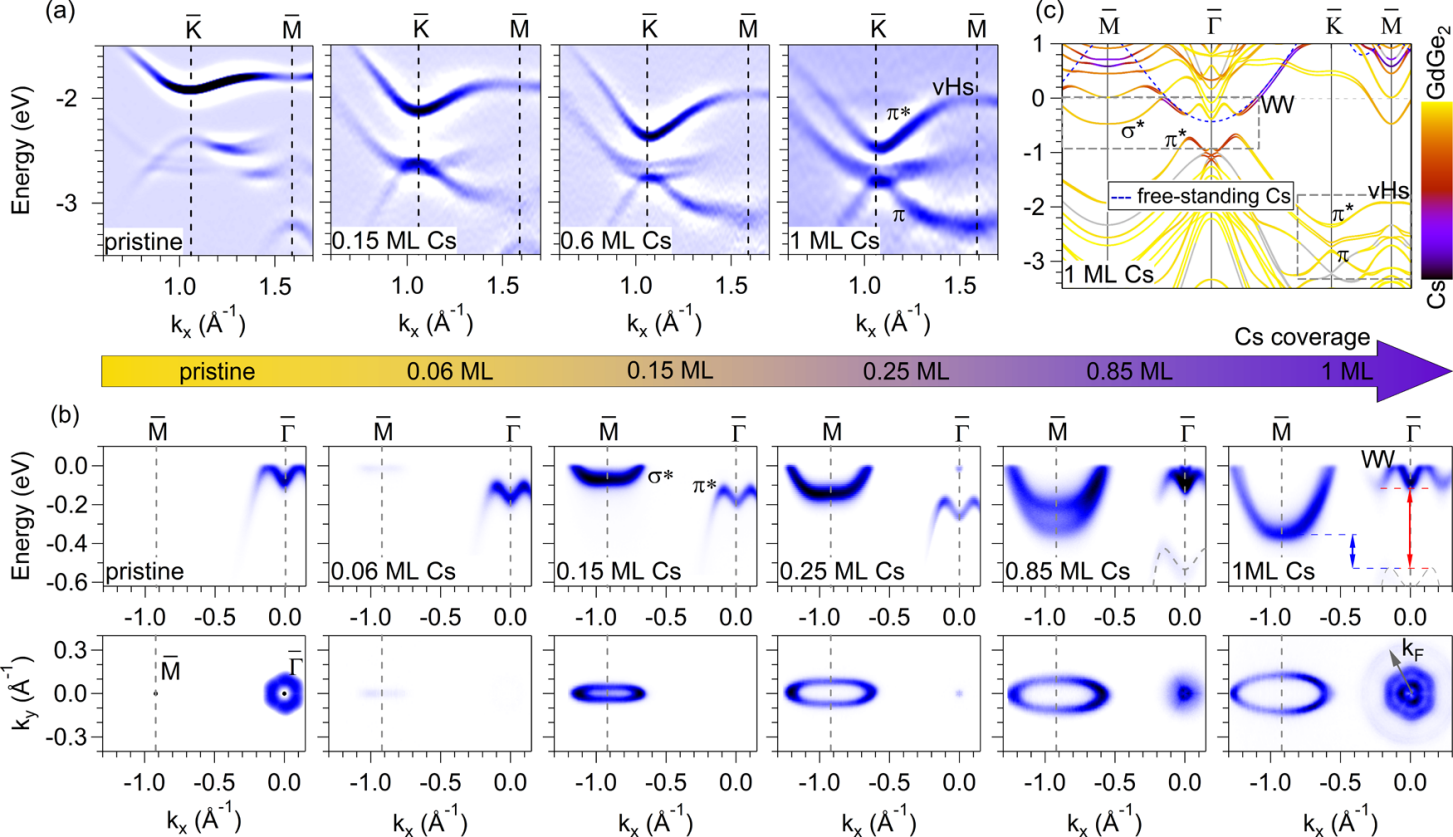
(a) ARPES spectra along K̅-M̅ as a function of the Cs doping with a focus on the Dirac cone and vHS. The data are shown as the second derivative of the photoemission intensity with respect to the energy. (b) ARPES spectra along M̅-Γ̅ (top row) and corresponding Fermi surfaces (bottom row) as a function of Cs doping. Horizontal dashed blue lines in the rightmost subpanels (1 ML Cs) mark the π*−σ* indirect gap (blue arrow); the dashed red lines and arrow show the hybridization gap at Γ̅. *k*_*F*_ marks the Fermi vector of the electron pocket related to the 
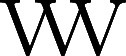
 band. All ARPES data are taken at 14 K with 35 eV photon energy. (c) DFT spectrum of the nonmagnetic GdGe_2_/Ge­(111) case with Cs on the surface. Gray color shows predominantly the Ge substrate bands, and the gradient lines changing a color from yellow to violet exhibit relative weights of GdGe_2_ and Cs orbitals, respectively. Dashed blue line shows the low-energy band of the spectrum of the free-standing Cs monolayer. Dashed-line gray rectangles highlight the *E*(*k*_∥_) areas shown in ARPES panels.

The Dirac cones and vHSs reported above are the key electronic properties of a honeycomb lattice. Their ARPES observation supported by DFT together with a nonzero local spin-Berry curvature confirm that GdGe_2_ is indeed a germanene-like system. Due to the high binding energy, these Dirac states may be less relevant for spintronics. On the other hand, the same large mixing of the Gd-*d* orbitals with germanene states brings to the proximity of *E*_F_ other singularities that have not been addressed yet in experiments. Toward Γ̅, the π* band forms a two-humped-shaped band that crosses *E*_F_ and determines the Fermi surface of the pristine GdGe_2_ system (Figure 2b, leftmost subpanels). Just above *E*_F_ a highly anisotropic band resides at M̅: it is almost flat along M̅ – Γ̅ and strongly dispersing along M̅ – K̅ (Figure 1d). Although the dispersion of this state resembles the σ* band of the free-standing germanene,^28,29^ its orbital composition is primarily determined by the Gd-*d* orbitals (Supplementary Figure S7). An indirect gap of about 0.2 eV separates the top of the two-humped π* and the bottom of the unoccupied σ* state in the calculated spectrum (Figure 1d). The system therefore can be easily tuned from pristine *p*-doped to semiconducting and then to *n*-doped by Cs doping. Figure 2b shows the effect of Cs doping on the band structure along Γ̅-M̅ (top row) and on the Fermi surface (bottom row). Our pristine GdGe_2_ sample happened to be *p*-doped with −0.02 electrons per unit cell. At about 0.06 ML Cs dose, the π* band becomes fully occupied and there are no states at *E*_F_, except for a very low intensity at M̅ derived from the broadening of σ*. Correspondingly, we observed moderate charging effects during photoemission experiments (Supplementary Figure S8), confirming the presence of a π*−σ* gap. With increasing Cs dose, the σ* state becomes occupied producing elongated electron pockets on the Fermi surface, turning the system into a metal. For higher Cs dose, the shape of the σ* state is modified – it becomes more parabolic-like. We notice the appearance of a splitting of the σ* state at 0.85 ML of Cs, that disappears with further doping. The origin of the appearance/disappearance of the splitting will be discussed below. Above about 0.25 ML Cs, additional states become visible at Γ̅, which evolve into a 
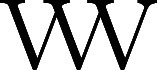
-shaped band (when approaching 1 ML of Cs) separated from a weakly intense two-humped band below by a gap of about 0.4 eV (Figure 2b).

In order to understand the changes in the spectrum near *E*_F_ upon increasing the Cs dose from low to high coverage, we refer to the calculated spectrum of the GdGe_2_/Ge­(111) film with 1 ML of Cs on the surface (Figure 2c). The spectrum shows, as expected, the presence of the σ* band in the occupied part at M̅ as well as the 
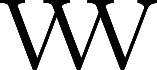
-shaped band at Γ̅. Herewith, the outer branches of the latter state are localized on the Cs monolayer. The weight of Cs orbitals also dominates the new deep two-humped state at ≈ −1 eV. Comparing the spectrum with the band dispersion of the band in the free-standing Cs ML (dashed blue line) one can conclude that the large gap observed at Γ̅ at high Cs coverage stems from the hybridization between the Cs band (of *s* character) and the π* state of the GdGe_2_ monolayer.

### Manipulation of Magnetic Order in GdGe_2_

Due to the strong contribution from the Gd orbitals, the GdGe_2_ monolayer is expected to manifest magnetic properties. The bulk GdGe_2_ material is reported to be antiferromagnetic, with an in-plane FM interlayer interaction and an AFM intralayer coupling.^36^ This ground state was confirmed down to double layers of GdGe_2_ by recent studies that found an experimental band splitting in agreement with the DFT prediction for an AFM order.^21,24^ Regarding the magnetic order of the GdGe_2_ monolayer, there is a discrepancy in the literature: DFT studies reported an in-plane FM ground state,^21,24^ while the experimental SQUID and XMCD data on SiO_*x*_ capped GdGe_2_ 1 ML film suggested a more complicated spin arrangement^20,23^ with a rather low magnetic moment explained as a competition of FM and AFM orders.

Our ARPES data for the pristine GdGe_2_ monolayer did not show any indication of a band splitting down to the lowest temperature of our experimental setup (about 14 K). In order to avoid the possible effects of the capping layer, we performed XMCD studies at the Gd M_4,5_–edges on an in situ grown GdGe_2_ monolayer film. We found that the magnetization curves did not show hysteresis down to 1.8 K and did not saturate up to 6 T for both in-plane and out-of-plane field directions (Figure 3a). We can estimate the maximal value close to 2.5 μ_B_/atom, that is reduced as compared to about 7 μ_B_/atom of a saturated FM Gd layer (Supplementary Figure S9). From the temperature-dependent hysteresis data, we extracted the low-field magnetic susceptibility, which follows the Curie–Weiss law with a clear negative temperature offset θ_*N*_ = −14 K (Figure 3b), a value in qualitative agreement with the SiO_*x*_ capped films.^20^ These results indicate an AFM coupling with a small in-plane anisotropy. We notice the absence of an upward bending or a flattening of the inverse magnetic susceptibility at low temperatures, which should signal an AFM transition, and the magnetic moments that do not saturate even in the 6 T magnetic field. Together with the absence of any visible band folding in the ARPES data, these findings indicate a short-range AFM order and/or a very low transition temperature.

**Figure 3:**
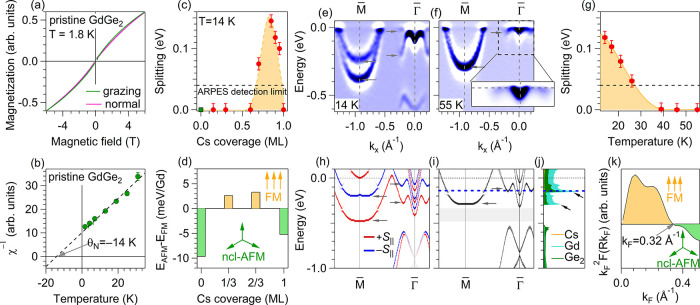
(a) Magnetization curves measured at the Gd M_5_ edge (1188.9 eV) with grazing (green) and normal (violet) incident light at 1.8 K, showing the absence of hysteresis. (b) Inverse magnetic susceptibility χ^–1^ vs temperature *T*, extracted from XMCD data at magnetic field *H* = 0.3 T. (c) Energy splitting of the electron band at M̅ as a function of Cs coverage at 14 K. (d) Calculated relative energy difference of the Cs/GdGe_2_/Ge­(111)- $\left(\sqrt{3}\times \sqrt{3}\right)$ structures between noncollinear antiferromagnetic (ncl-AFM) and ferromagnetic (FM) coupling on the Gd sublattice with Cs coverage $\Theta =0,\frac{1}{3},\frac{2}{3}$ , and 1 ML. (e) ARPES data for 0.85 ML Cs/GdGe_2_ film (e) at 14 K and (f) at 55 K along M̅ – Γ̅. The data are shown as the second derivative of the photoemission intensity with respect to the energy. The inset shows the zoom on Γ̅ for prisine data. (g) Energy splitting of the electron band at M̅ as a function of temperature for 0.85 ML Cs/GdGe_2_. (h) DFT spectrum for the FM_∥_ state with 0.66 ML of Cs on the surface (two Cs atoms per $\left(\sqrt{3}\times \sqrt{3}\right)$ cell) unfolded onto the (1 × 1) surface Brillouin zone of GdGe_2_. (i) The same as (h) for nonmagnetic case. Gray arrows point spin splitting in (e) and (h) and its missing in (f) and (i). (j) Element-resolved DOS for nonmagnetic calculation. Blue dashed line in (i) and (j) marks the position of the experimental *E*_F_. (k) Oscillatory term of the RKKY interaction as a function of Fermi vector.

We performed the magnetic ground state calculations and found that indeed a noncollinear AFM order with 120° spin arrangement in the Gd plane is energetically much more (about 10 meV/Gd atom) favored than the FM one (Figure 3d, zero coverage limit). The calculated band structure of the ncl-AFM phase completely coincides with the that of the nonmagnetic case (see Supplementary Figure S10). Thus, the magnetic phase transition toward an ncl-AFM state would not be observable in ARPES.

As noted earlier, at about of 0.85 ML Cs coverage (Figure 2b) a sizable band splitting is observed at M̅. Figure 3c shows a detailed dependence of the splitting of the σ* band upon Cs coverage in the range from 0.15 to 1 ML. For Cs coverages above 0.6 ML, the splitting increases (the splitting detection limit is about 0.04 eV, indicated as a horizontal dashed line in Figure 3c), reaching a maximum value of 0.14 eV at about 0.85 ML (Figure 3e) and then decreases to zero at 1 ML coverage. This splitting gradually vanishes as the temperature increases (Figure 3g), approaching zero at *T* ≈ 35 K (and remains so at higher temperatures, Figure 3f) proving its magnetic origin. The magnetic splitting is observed likewise in the 
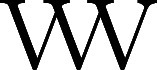
-shaped band (Figure 3e). At a Cs coverage of 0.85 ML it is also maximal and reaches 0.19 eV (Supplementary Figure S11).

Our DFT calculations for the structures with an unsaturated Cs ML (with 1 and 2 Cs atoms per $\sqrt{3}\times \sqrt{3}$ supercell, corresponding to Cs coverages of 0.33 and 0.67 ML, respectively) show that the ncl-AFM phase becomes unfavorable compared to FM configurations (see Figure 3d), irrespective of in-plane/out-of-plane spin alignment. Note that the magnetic anisotropy in the FM configurations is negligibly small (with a small preference for the in-plane FM). However, at a saturated monolayer (3 Cs atoms per $\sqrt{3}\times \sqrt{3}$ supercell), ncl-AFM again becomes preferable. Such a change of the magnetic behavior of the GdGe_2_ with an increase of Cs coverage is in line with the observed Θ-dependence of the σ* and 
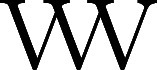
 bands splittings. The calculated band structures for in-plane FM and nonmagnetic cases for 0.66 ML Cs (Figure 3h,i) are in good agreement with our ARPES data for low and elevated temperatures (Figure 3e,f).

The observed switching of the magnetic ground state in the GdGe_2_ monolayer from AFM to FM with a variable splitting and, the most surprising, back to AFM at a full 1 ML Cs coverage can be qualitatively explained by the following formula for the RKKY interaction in 2D free electron gas systems^37^:

$$\begin{array}{c} j^{2D}(Rk_{F})=-\frac{J^{2}m^{*}k_{F}^{2}}{8(\pi n\hbar {)}^{2}}F(Rk_{F})\\ F(Rk_{F})=J_{0}(Rk_{F})N_{0}(Rk_{F})+J_{1}(Rk_{F})N_{1}(Rk_{F}) \end{array}$$

where *R* is the distance between the magnetic atoms that is equal to the lattice constant, *J* is the interaction constant, and *n* is the density of the magnetic atoms. The conduction electrons are presented here through the effective mass m*, the Fermi vector *k*_F_, and through the oscillatory function *F* that combines Bessel functions of the first (*J*_*n*_) and second (*N*_*n*_) kinds.

The dependence of the oscillatory part of the RKKY interaction, *k*_F_^2^ *F*, on the Fermi vector *k*_F_ is shown in Figure 3k. We can see that it remains positive between *k*_F_ = 0 and *k*_F_ = 0.32 Å^–1^ (Supplementary Figure S12). The observed magnetic transitions can be understood as follows: (1) The pristine GdGe_2_ monolayer is slightly *p*-doped (m* < 0), with a single hole pocket at Γ̅ having an effective *k*_F_ below 0.1 Å^–1^. Overall, it results in a positive *j* ^2D^ and in AFM correlations. (2) Above 0.15 ML Cs doping, the system becomes *n*-doped (m* > 0), so *j* ^2D^ < 0 and the FM order starts to be more favorable. The same transition can be observed in DFT for artificially electron-doped GdGe_2_ monolayer (Supplementary Figure S13). Due to a strong anisotropy of the oval pockets at M̅, the m* term is not large, resulting in a low *j* ^2D^, low *T*_C_ and in a band splitting not observable in ARPES. (3) Above 0.7 ML (Supplementary Figure S11) the 
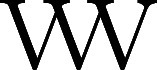
-shaped band with Gd *d* character starts to grow rapidly. Its wavy dispersion leads to a high m*, as can also be seen from the peak in the DOS close to *E*_F_ (Figure 3j). This results in an increase of the *T*_C_ and in a sizable splitting observed in ARPES. (4) Close to 0.95 ML, the *k*_F_ of the 
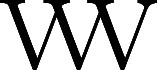
-band reaches 0.33 Å^–1^, leading to a negative *k*_F_^2^ *F* term and hence to a positive *j* ^2D^ and to the AFM order. We note that similar transitions as a function of *k*_F_ were predicted for 2D magnetic materials but so far not observed experimentally.^16,17,30^

## Conclusions

We performed a comprehensive study of the electronic and magnetic properties of the germanene-like buckled GdGe_2_ monolayer grown on a bulk germanium substrate. The absence of surface reconstruction allows for unambiguous identification of the Dirac cones and van Hove singularities related to the germanene honeycomb lattice, both of which lie within the substrate gap. Due to the hybridization with Gd, the Dirac cone shows a wide gap reaching 0.5 eV, which can be largely and reversibly tuned by electron doping. We confirm the topological properties of the gap by a nonzero spin-Berry curvature in the proximity of the Dirac point. Large electron doping shifts previously unexplored singularity states—peculiar to germanene—toward the Fermi level, enabling to drive to drive the system across different metal–semiconductor transitions. We furthermore demonstrate that change in the electron occupation of these states induces multiple magnetic transitions. The pristine monolayer has antiferromagnetic correlations, as shown by experimental observation and theoretical calculations. Under controlled cesium doping, it turns into a ferromagnet with a tunable Curie temperature, and into antiferromagnet at saturation doping. We explain this unusual behavior by the oscillatory behavior of the RKKY interaction as a function of the Fermi wave vector, predicted for 2D materials.

We suggest that such a rich band structure opens a pathway for the realization of different phase transitions in GdGe_2_. Because of a semiconducting substrate, the magnetic state can be switched from ferromagnetic to antiferromagnetic and vice versa by an electric gating. The large exchange splitting of electron doped samples may allow the realization of half-metallic states, as well as lead to a metal–semiconductor transitions as a function of temperature. The strong two-dimensional confinement of the electrons and high effective mass of the σ* and π* states at *E*_F_ can lead to an enhancement of the correlation effects resulting in spin- and charge-density waves and quasiparticle excitations. The magnetic properties of the materials can be further engineered by the rare earth metal substitution, which form isostructural phases with Ge but have different magnetic moments.

## Methods

### Sample Preparation

The GdGe_2_ monolayer was grown in situ. The Ge(111) substrates were sputtered with Ar^+^ ion bombardment and then annealed at 650 °C; this procedure was repeated several times until a sharp *c*(2 × 8) LEED pattern was obtained. Gd was deposited on the Ge(111) substrate and annealed to about 500 °C. For 1 ML coverage this procedure results in a 1 × 1 LEED in agreement with previous studies, without additional $\sqrt{3}\times \sqrt{3}-R30^{{}^{\circ}}$ pattern that can be observed for thicker layers.^21,24^ The cesium coverage was calibrated by the deposition time (1 ML was determined from saturation of the electronic band structure) and cross-checked at the intermediate steps from $\sqrt{3}\times \sqrt{3}-R30^{{}^{\circ}}$ (0.6–0.66 ML) and $\sqrt{7}\times \sqrt{7}-R19.1^{{}^{\circ}}$ (0.87 ML) LEED reconstructions (Supplementary Figure S14).

### Photoemission Measurements

Angle-resolved photoemission spectroscopy (ARPES) data were taken at the VUV-Photoemission beamline of the Elettra synchrotron (Italy). Measurements were performed using a Scienta R4000 electron analyzer and excitation energies between 25 and 155 eV with linearly polarized light. The electron spectrometer was placed at 45° with reference to the direction of the incoming photon beam. The base pressure of the analytic and preparation chambers was ≤1.0 × 10^–10^ and ≤3 × 10^–10^ Torr, respectively.

### Magnetic Measurements

X-ray magnetic circular dichroism (XMCD) measurements of the GdGe_2_ monolayer were made in situ at the DEIMOS beamline of the SOLEIL synchrotron (France) with magnetic fields up to 6 T and at temperatures down to 1.8 K. Circularly polarized X-rays were incident upon the sample at 0 and 70° from normal, with a magnetic field applied parallel to the X-ray beam. Detection was performed using surface-sensitive total electron yield mode.^38,39^

### DFT Calculations

The calculations were based on density functional theory (DFT) as implemented in the Vienna Ab initio Simulation Package VASP.^40^ The projector-augmented wave approach^41^ was used to describe the electron–ion interaction and the generalized gradient approximation (GGA) of Perdew, Burke, and Ernzerhof (PBE)^42^ was employed as the exchange-correlation functional. The scalar relativistic effect and the spin–orbit coupling (SOC) were taken into account. To simulate the GdGe_2_ structures we used a slab consisting of four bilayers (BL) of germanium with the PBE-optimized bulk lattice constant (4.09 Å). Hydrogen atoms were used to passivate the dangling bonds at the bottom of the slab. The atomic positions of the adsorbed Gd atoms and the atoms of the upper Ge layer as well as the Ge atoms within the three upmost BLs of the slab were optimized (Supplementary Figure S1). The substrate atoms of the fourth layer were kept fixed at the bulk crystalline positions. The kinetic cutoff energy was 250 eV, and a 12 × 12 × 1 *k*-point mesh was used to sample the 1 × 1 supercell BZ, respectively. The geometry optimization was performed until the residual force on atoms was smaller than 10 meV/Å. For band-structure calculations, two types of Gd pseudopotentials were used.^43^ The Gd potentials, where strongly localized, valence 4*f* electrons are treated as core states, were used for nonmagnetic band-structure calculations. In order to describe the magnetic properties, standard Gd potentials were used for spin-polarized noncollinear calculations, in which *f* electrons are treated as valence states.^44^ The Heyd-Scuseria-Ernzerhof (HSE06) screened hybrid functional was used to accurately calculate the Ge gap and to avoid the self-interaction errors arising from an incorrect description of the partially filled *f* states of Gd.^45^ However, in supercell calculations a Hubbard GGA + *U* approach within the Dudarev method^46^ was used. The *U*_eff_ = *U* – *J* was chosen to be equal to 7.0 eV. The unfolding of the band structure was performed using the BandUP code.^47,48^ Wannierization procedure of the DFT-derived spectra and following derivation of the Hamiltonians written in the Wannier functions basis were performed using the Wannier90 code.^49^ For the *k*-resolved spin Berry curvature calculations, the method proposed by Ryoo et al.^50^ for calculating the spin velocity matrix was utilized within the WannierBerri code.^51^ For the *n*-th band the spin Berry curvature Ω_*xy*_^*n*,*z*^ is defined as

Ωxyn,z(k)=−∑m≠n2Im[⟨nk|jxz|mk⟩⟨mk|υy|nk⟩](ϵnk−ϵmk)2−(iη)2

where *j*_*x*_^*z*^ and υ_*y*_ are the spin current and velocity operators respectively, and η is a fixed smearing parameter (0.05 eV).

## Supplementary Material



## Notes

Structural model of GdGe_2_ with and without Cs; ARPES: spectra of clean GdGe_2_ at high temperatures, of clean Ge(111) surface, of deeply lying Gd *f*-states and as a function of time and annealing; LEED: diffraction patterns of different Gd- and Cs-induced reconstructions; XMCD: detailed XMCD spectra; and DFT: orbital analysis of the band structure of GdGe_2_, artificial hole doping of GdGe_2_, comparison of band structures corresponding to different magnetic orders (PDF)
