# Supplementary material for: Germanene-Based Two-Dimensional Magnet with Tunable Properties
Source: ACS Nano. 2025 May 29;19(22):20863–70. doi: 10.1021/acsnano.5c03331 (PMC12164529; doi:10.1021/acsnano.5c03331)
Supplement: Supplementary file 1 [file nn5c03331_si_001.pdf]

Supplementary Material for: Germanene-Based Two-Dimensional Magnet with Tunable Properties

Andrey V. Matetskiy,[1,*] Alessandro Barla,[1] Paolo Moras,[1] Carlo Carbone,[1] Valeria Milotti,[1,†] Carlo Alberto Brondin,[1] Zipporah Rini Benher,[1] Mariia Holub,[2] Philippe Ohresser,[2] Edwige Otero,[2] Fadi Choueikani,[2] Igor A. Shvets,[3] Alexey N. Mihalyuk,[4,5] Sergey V. Eremeev,[6,7] and Polina M. Sheverdyaeva[1,‡]

[1] *CNR-Istituto di Struttura della Materia (CNR-ISM), Strada Statale 14, km 163.5, 34149 Trieste, Italy*

[2] *Synchrotron-SOLEIL, L'Orme des Merisiers, 91190 Saint-Aubin, France*

[3] *Tomsk State University, Tomsk 634050, Russia*

[4] *Institute of High Technologies and Advanced Materials, Far Eastern Federal University, Vladivostok 690950, Russia*

[5] *Institute of Automation and Control Processes FEB RAS, Vladivostok 690041, Russia*

[6] *Institute of Strength Physics and Materials Science SB RAS, Tomsk 634055, Russia*

[7] *St. Petersburg State University, 7/9 Universitetskaya nab., St. Petersburg 199034, Russia*

* Present affiliation: Peter Grünberg Institute (PGI-3), 52428 Forschungszentrum Jülich, Germany

† Present affiliation: Dipartimento di Fisica e Astronomia "Galileo Galilei", Università degli Studi di Padova, 35122 Padova, Italy

‡ polina.sheverdyaeva@trieste.ism.cnr.it

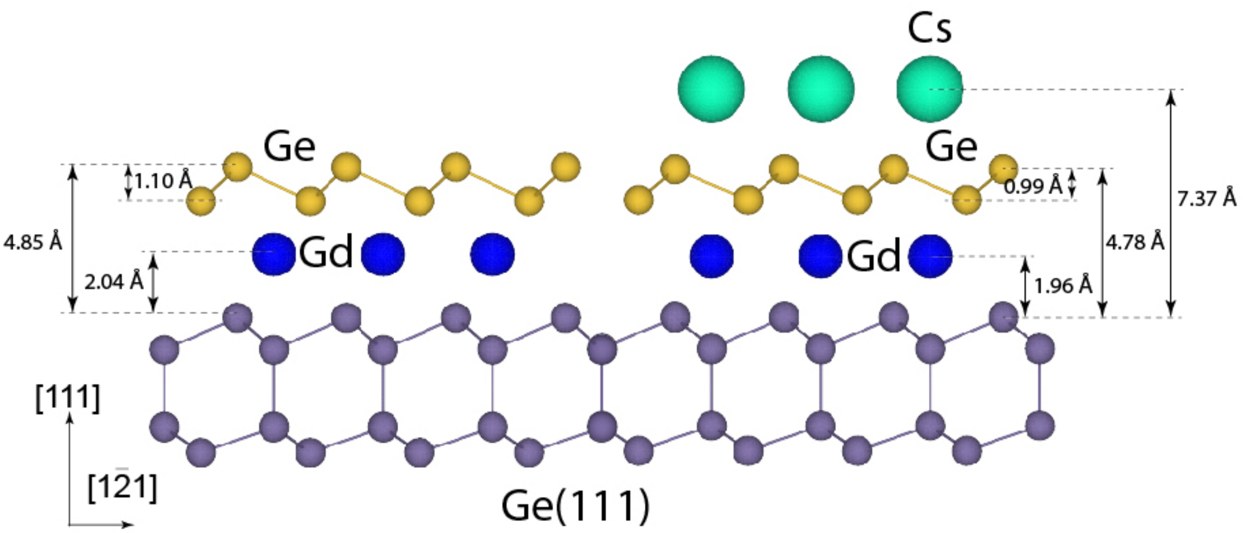

FIG. S1. **Structural characteristics of pristine and Cs covered $GdGe_2$ film.** Structural characteristics of the $GdGe_2$/Ge(111) film at pristine state (left-side) and after 1 ML Cs adsorption (right-side). The most energetically favorable position for Cs atoms is above Gd atoms in the voids of the germanene honeycombs. The Ge-Ge bonds length in germanene monolayer decreases from 2.60 Å to 2.56 Å due to the adsorption of Cs adatoms, while the buckling height decreases from 1.10 Å to 0.99 Å. The Cs-Ge bonds length is 3.50 Å.

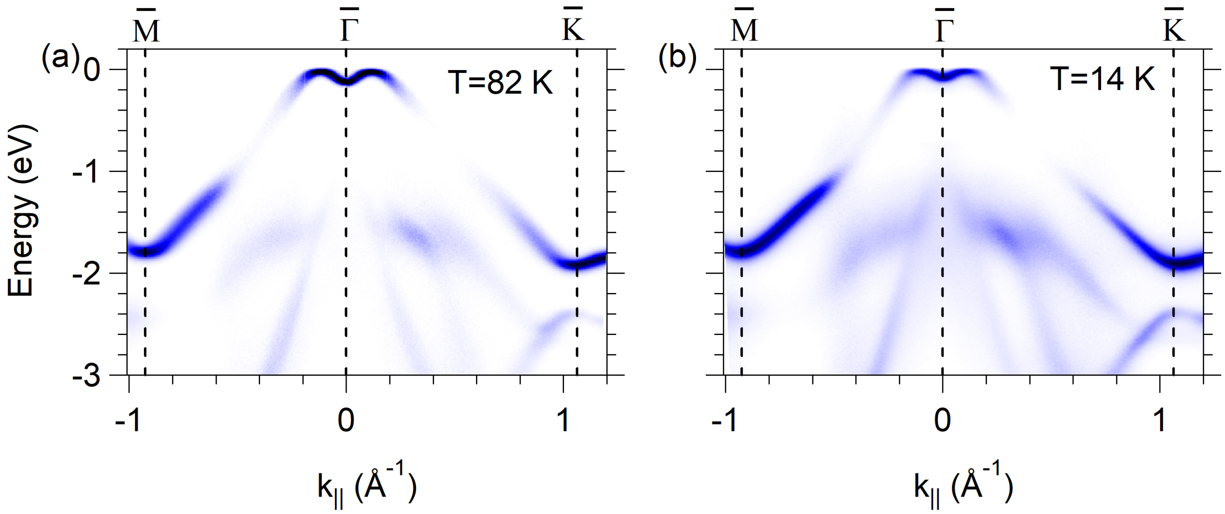

FIG. S2. **Temperature-dependent evolution of electronic spectra of pristine $GdGe_2$ film.** ARPES spectra recorded at 82 K (a) and 14 K (b) temperature, respectively.

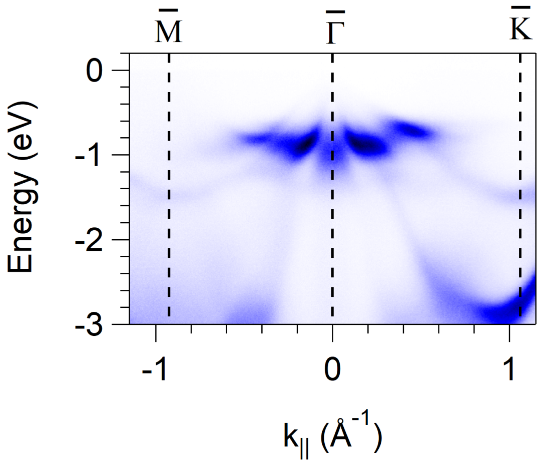

FIG. S3. **ARPES characterization of the clean Ge(111) substrate.** ARPES spectrum of clean Ge(111) substrate taken at 14 K with 35 eV photon energy.

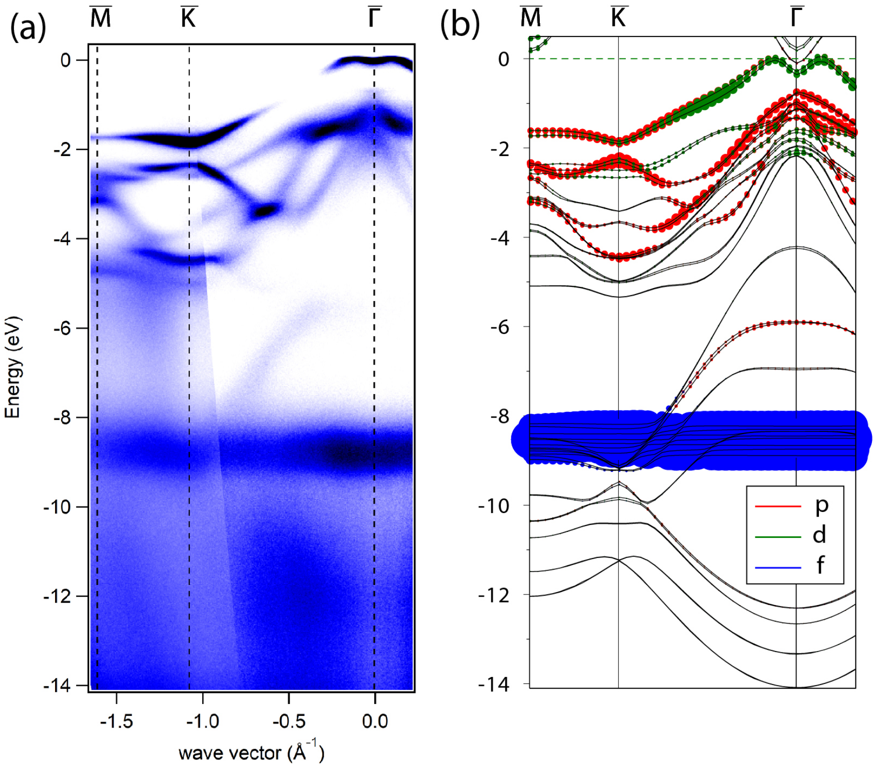

FIG. S4. **Experimental and calculated positions of Gd** $4f$ **states in** $\mathbf{GdGe_2}$**/Ge(111).** (a) Large-energy scale ARPES spectrum demonstrating the position of Gd-f band (spectrum is taken at 14 K, with 35 eV photons). (b) Electronic band structure calculated within HSE06 with weights of Gd orbitals marked by different colors.

.

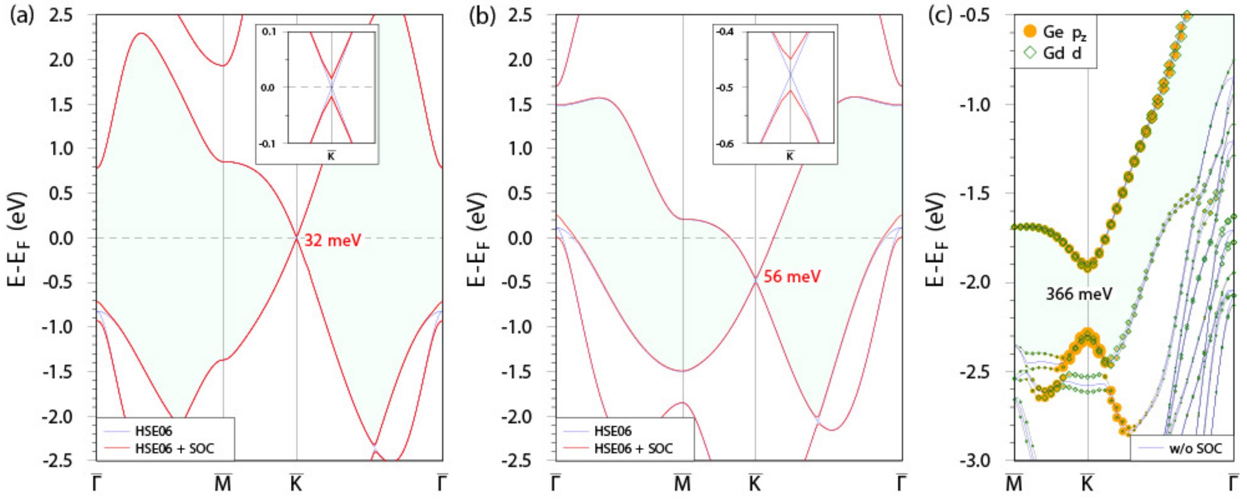

FIG. S5. **The effect of buckling and substrate on the germanene electronic band structure.** (a) DFT spectrum of free-standing equilibrium germanene calculated without and with SOC. (b) The same for the buckled Ge bilayer as it split off the Gd/Ge substrate. Insets in (a) and (b) show magnified view of the spectra at $\overline{\mathrm{K}}$. (c) Orbital projected states in $GdGe_2$/Ge(111) in the vicinity of the $\overline{\mathrm{K}}$ point gap. Blue lines show the spectrum calculated without SOC.

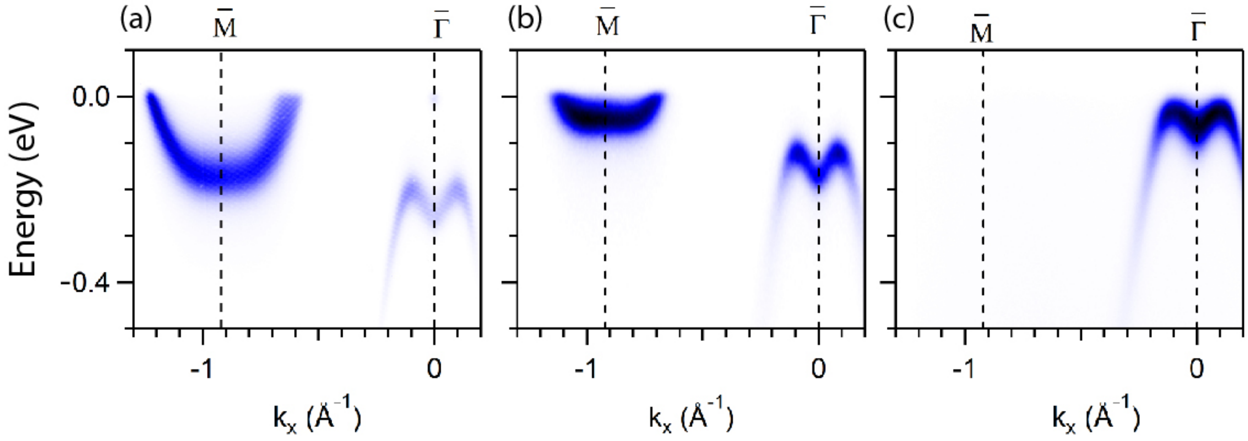

FIG. S6. **The effect of annealing on the electronic band structure.** ARPES spectra of progressively annealed Cs+$GdGe_2$ sample showing reversibility of the doping effect. (a) 0.2 ML Cs; (b) anneal at 400 K for 3 min; (c) anneal at about 773 K for 4 min.

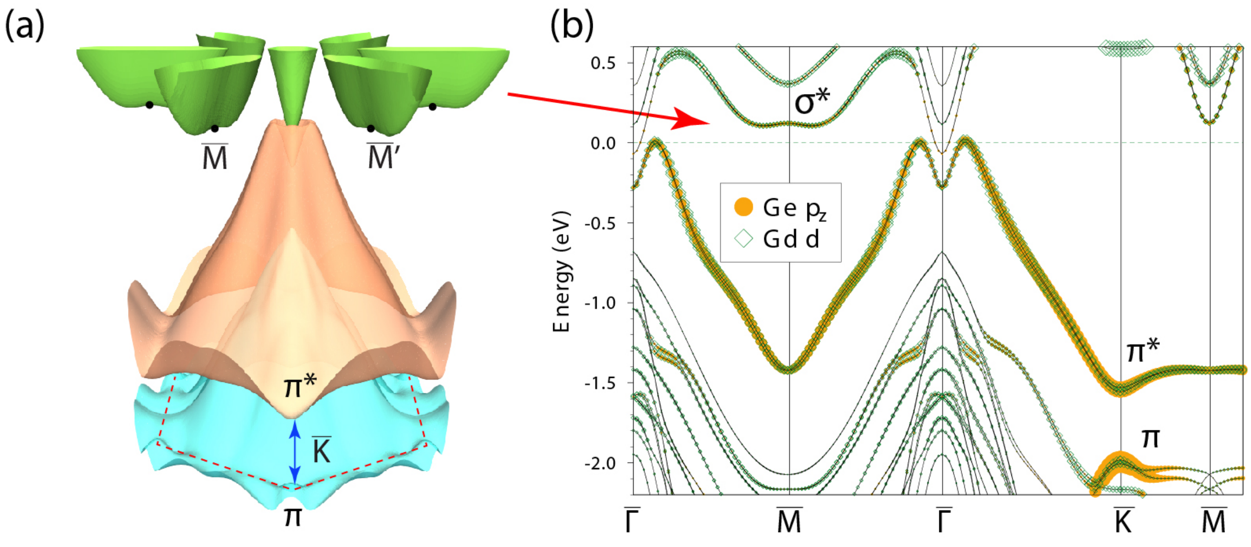

FIG. S7. **The character of germanene-like bands.** (a) Electronic structure of nonmagnetic $GdGe_2$ calculated on the surface Brillouin zone (three bands closest to E_F are shown) and (b) its element- and orbital-projected spectrum plotted along high-symmetry directions.

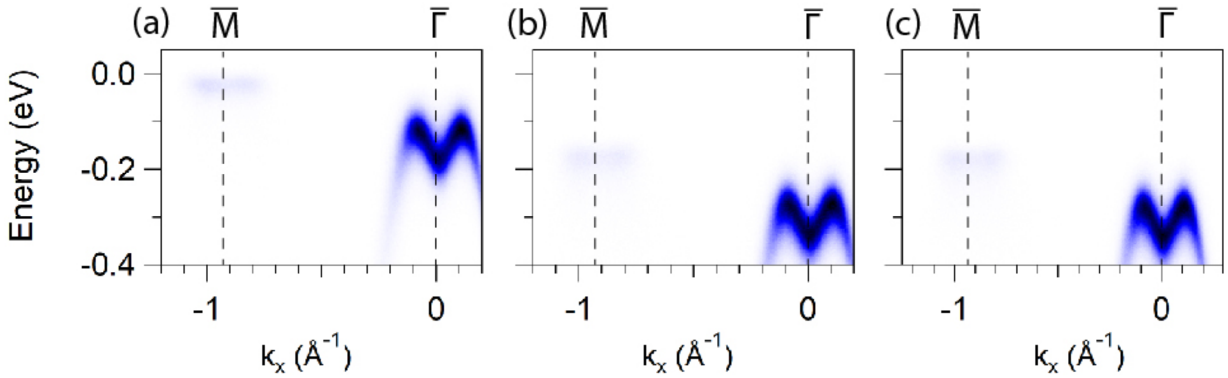

FIG. S8. **The effect of charging on the ARPES spectra.** ARPES spectra taken as a function of time on 0.07 ML Cs doped sample (a) just after exposure to synchrotron light, (b) after 3 min exposure and (c) after 6 min exposure. The Fermi level is referred to Mo plate in a contact with the sample.

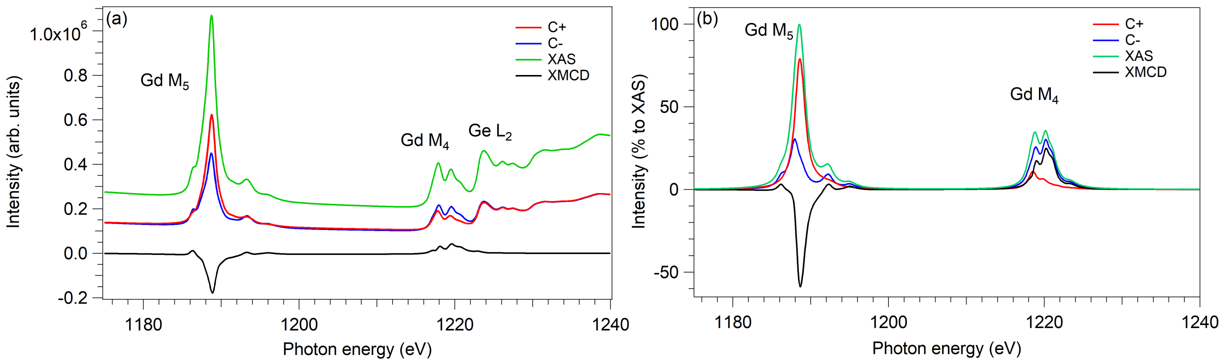

FIG. S9. **XMCD of pristine $GdGe_2$ film.** (a) XMCD and x-ray absorption spectroscopy (XAS) signals at the Gd $M_{4,5}$ edge taken at 1.8 K with 6 T field, grazing beam/field incidence direction. (b) Calculated XMCD spectrum for a free Gd^{3+} ion, corresponding to a total magnetic moment of 6.92 μ_B, obtained with the multiX code [1] for 1.8 K and 6 T. The intensity is normalized to the maximal XAS signal. In the experimental spectra, the overlap with Ge L_2 edge does not allow to use properly the sum rules and the determination of the absolute magnetic moment values. However, from the comparison of the relative experimental XMCD signal (about 21 % as of the XAS step at Gd M_5 edge) to the calculated one (about 58 %, panel (b)) , we can estimate the maximal value close to 2.5 μ_B/atom. This value is reduced as compared to a saturated FM Gd layer, but would be too large for a collinear AFM order. On the other hand, it is in line with a ncl-AFM correlation supported by the negative temperature offset $\theta_N = -14$ K in the Curie-Weiss law and by our DFT calculations.

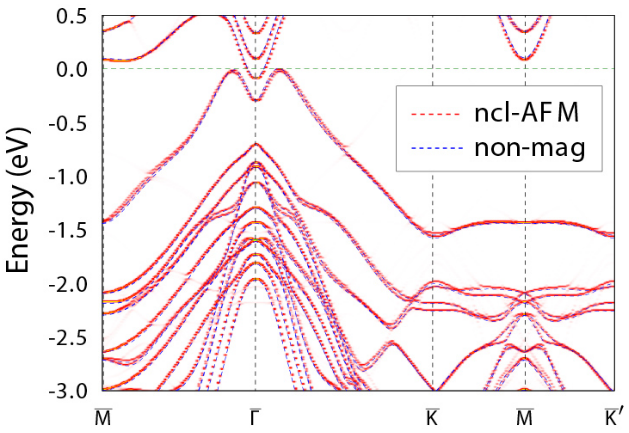

FIG. S10. **The influence of noncollinear AFM ordering on the electronic band structure.** The comparison of noncollinear AFM-120° (red dashed curves) and nonmagnetic (blue dashed curves) band structures calculated within PBE+U approach.

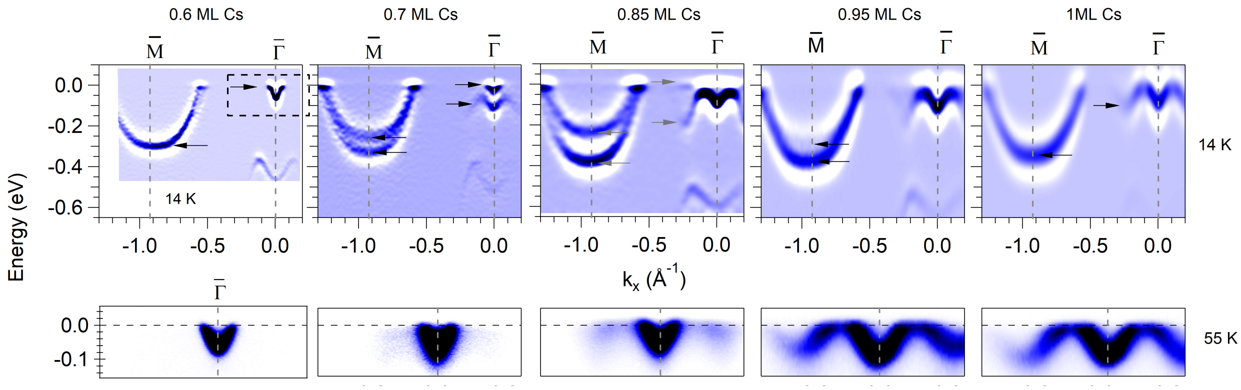

FIG. S11. **The evolution of $GdGe_2$ band structure on Cs doping.** Top row: ARPES spectra along $\overline{\mathrm{M}}$-$\overline{\Gamma}$ as a function of Cs doping, taken at 14 K, second derivative along the energy axis; bottom row: zoom on $\overline{\Gamma}$ taken at 55 K, except for the 0.6 ML Cs where it has been taken at 14 K.

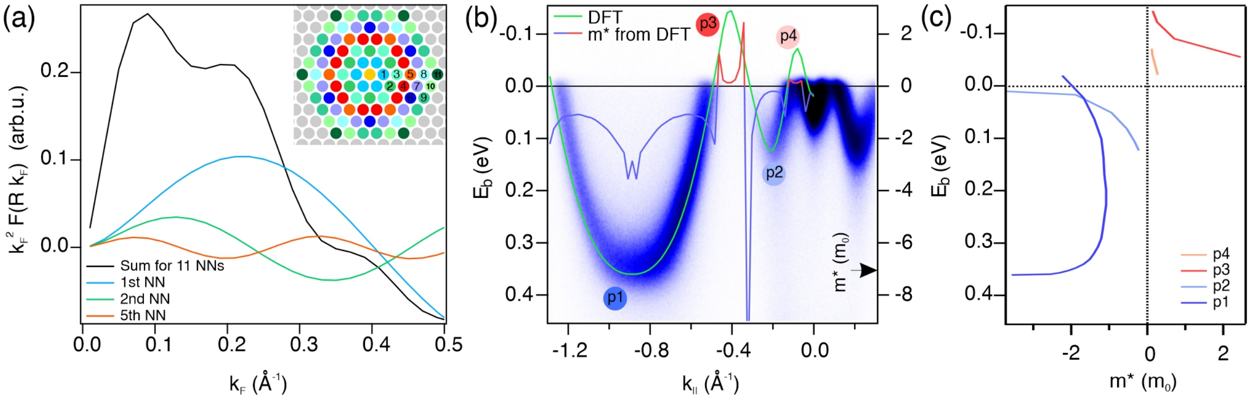

FIG. S12. **The behavior of the constituent terms of the RKKY equation versus Fermi vector.** (a) Oscillatory term of the RKKY interaction as a function of Fermi vector for selected nearest neighbors (NNs) and the sum for the first eleven of them (see the scheme in the inset). The experimental lattice constant was used to calculate corresponded Rs. (b) Observed band structure of the $GdGe_2$ monolayer with the 1 ML Cs. The corresponded DFT band structure is superimposed onto the ARPES spectrum. The dispersion provided by DFT was used in order to obtain the effective mass that is also shown. (c) Dependence of the effective mass on the band filling derived from (b). The axis are rotated in order to match the dispersion picture. P1-P4 marks the various pockets that may participate in the RKKY interaction. Blue color corresponds to the electron pockets, red to the hole pockets.

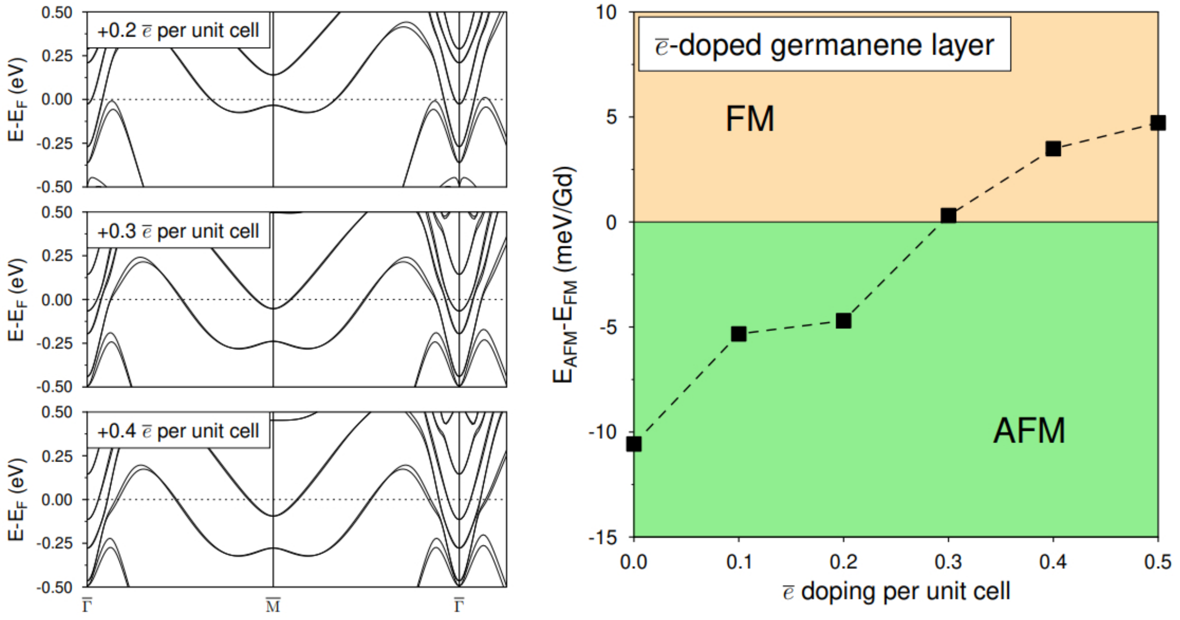

FIG. S13. **The effect of electronic doping on AFM-FM magnetic transition.** Left side: Evolution of $GdGe_2$ band structure in nonmagnetic phase on electronic doping of germanene monolayer. Right side: phase diagram demonstrating the magnetic order as a function of doping. The electron doping was simulated using the virtual crystal approximation by mixture of Ge and As potentials for atoms of germanene layer.

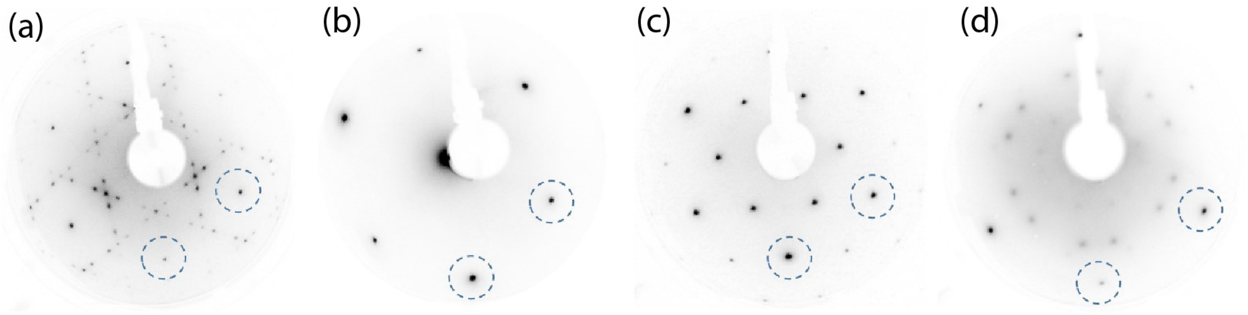

FIG. S14. **Evolution of LEED pattern at different coverages of Cs.** LEED spectra corresponding to: (a) clean Ge $c(2 \times 8)$, (b) clean $GdGe_2$ (1×1), (c) 0.66 ML of Cs on $GdGe_2$ $(\sqrt{3} \times \sqrt{3} - R30°)$, (d) 0.86 ML of Cs on $GdGe_2$ $(\sqrt{7} \times \sqrt{7} - R19.1°)$. Dashed blue circles mark 1×1 spots.

[1] A. Uldry, F. Vernay, B. Delley, Systematic computation of crystal-field multiplets for x-ray core spectroscopies, Phys. Rev. B 85 (2012) 125133. doi:10.1103/PhysRevB.85.125133. URL https://link.aps.org/doi/10.1103/PhysRevB.85.125133
